# Participant views and experiences of sexual health research: The *Contraception Choices* online trial

**DOI:** 10.1177/20552076211033424

**Published:** 2021-08-12

**Authors:** Julia V Bailey, Kirsty F Bennett, Anasztazia Gubijev, Jill Shawe, Judith Stephenson

**Affiliations:** 1 e-Health Unit, Department of Primary Care and Population Health, 4919 University College London, UK; 2 Cancer Communication and Screening Group, Department of Behavioural Science & Health, 4919 University College London, UK; 3 4919 London School of Hygiene and Tropical Medicine, London, UK; 4 Faculty of Health, 6633 University of Plymouth, UK; 5 UCL Elizabeth Garrett Anderson Institute for Women’s Health, 4919 University College London, UK

**Keywords:** Digital health, eHealth, reproductive health, sexual health, women's health, qualitative

## Abstract

**Background:**

Online sexual health research can be convenient, efficient and low cost, but there are debates about the adequacy of online informed consent, privacy, and the acceptability of different methods of follow-up.

**Objectives:**

To explore women's views and experiences of the *Contraception Choices* feasibility trial procedures and the place of digital interventions for contraception decision making.

**Methods:**

We analysed data from two sources: (1) *Qualitative interviews*. Eighteen interviews were conducted with women who had taken part in the *Contraception Choices* pre-trial feasibility study, to evaluate recruitment and online trial procedures. (2) *Free-text comments*. Women in the main *Contraception Choices* randomised controlled trial were followed up at 3 and 6 months, and asked ‘Please tell us what you liked or disliked about the website’ and ‘Has being in the study had any good or bad effects on your life?’ A total of 387 and 414 comments were made at 3 and 6 months respectively. Data were analysed thematically.

**Results:**

Participants liked being involved in a study about contraception, although recruitment from an abortion clinic was less acceptable than in other sexual health settings. Women found the trial procedures straightforward, and expressed no major concerns about online self-registration, informed consent or online data collection. Online survey questions about contraception and fertility were acceptable, and participants liked the convenience of being followed up by email or text.

**Conclusions:**

Participants appreciated the advantages of the online research design and did not express concerns about consent or privacy. Women would welcome digital interventions for contraception in a variety of settings.

## Introduction

Digital technology offers huge potential for health promotion, and online interventions are particularly suitable for sexual health topics because access can be private, convenient and self-paced.^1^ Information about contraception is widely available online, but the quality is variable and the content can be misleading.^2,3^

The *Contraception Choices* website was developed in collaboration with young women, health professionals and a software company.^4^ The website provides information and support to help make informed choices about contraception. The site addresses the advantages and disadvantages of twelve different methods of contraception and takes into account women's preferences to provide three personally tailored contraception method suggestions which can be exported by email or text. The website features clearly presented text, videos of young women and health professionals talking about contraception, and interactive infographics (see Supplementary material 1 – Screenshots).

We evaluated the *Contraception Choices* website in an online trial.^5^ Online trials are relatively cheap, convenient for participants and can offer efficient data collection and automated follow-up.^6^ Sexual health data collected online may be more accurate than face-to-face data collection since privacy can facilitate more honesty.^7^ Online recruitment can be quick and easy, but, as with conventional trials, attrition can be large.^8–10^ Face-to-face recruitment and multiple modes of follow-up (e.g. email, text, telephone, post and clinic records) can help to reduce attrition.^11,12^

Although online research methods are not new, concerns are sometimes expressed by ethics committees and research sponsors about the adequacy of informed consent online, the sensitivity of sexual health questions, privacy and data protection and the acceptability of automated electronic follow-up.^13^ In this qualitative process evaluation, we aimed to explore women's views and experiences of the *Contraception Choices* online trial procedures to address these methodological uncertainties, and women's views on the place of digital interventions for contraception decision making.

## Methods

This study is a qualitative process evaluation of the *Contraception Choices* randomised controlled trial (RCT).

### Pre-trial feasibility study

Posters were placed in six clinic or community settings where contraception is offered (a general practice, sexual health clinic, antenatal clinic, abortion service, community pharmacy and a young person's sexual health clinic). Women aged 15–30 years were approached in the clinic waiting room by a researcher and invited to register for the study on a tablet computer. All feasibility trial participants were allocated to the *Contraception Choices* website in order to generate a cohort of women who could comment on the acceptability of the intervention. A follow-up online questionnaire was sent by email at 1-week post-randomisation, to test the trial procedures. Participants were asked whether they would be willing to be contacted for a post-study interview. Those who agreed were contacted by telephone and invited to participate in an interview 2 weeks later.

The main RCT design is described in Box 1, and trial outcomes are reported elsewhere.^5^

Box 1. Design of the online trial of the *Contraception Choices* website.

**Design:** Online randomised controlled trial

**Recruitment:** Posters were placed in six clinic or community settings where contraception is offered (a general practice, sexual health clinic, antenatal clinic, abortion service, community pharmacy and a young person's sexual health clinic), and female clinic attendees were approached in the clinic waiting room by research staff and invited to register for the study on a tablet computer.

**Eligibility:** Women aged 15 to 30 years with a need for contraception, able to read English and provide informed consent with an active email account and access to the internet.

**Online enrolment and consent:** Enrolment and consent for the study were sought electronically using a tablet computer with a researcher available to resolve any technical problems. Participants entered their contact details (email address and telephone number) online using the tablet computer.

**Baseline data:** Outcome data were collected online (demographic information and questions on fertility, contraception use, and satisfaction with the current contraception method).

**Randomisation:** Automated, computerised randomization with instant allocation to intervention or control group.

**Intervention:** Unlimited access to the *Contraception Choices* website with fortnightly emailed reminders to encourage engagement.

**Control:** Waiting list (access to the *Contraception Choices* website at the end of the study)

**Follow-up:** Participants were contacted by email 3 and 6-months post-randomisation and invited to click on a hyperlink to complete an online follow-up questionnaire that assessed contraception use, satisfaction with contraception method, health service use and quality of life. Two-free text questions were asked: ‘*What did you like or dislike about the website*’ and ‘*Has being in the study had any good or bad effects on your lif*e?’ Non-responders were prompted by email, text and telephone and a total of £20 in online shopping vouchers was offered for self-reported follow-up data.

**Trial registration number:** ISRCTN 13247829

**Ethical approval** was provided by the London – Camden & Kings Cross Research Ethics Committee (reference number 17/LO/0112)

### Data collection

This process evaluation analysed qualitative data from two sources:

1. *Qualitative interviews* with women who had taken part in the *Contraception Choices* pre-trial feasibility study.
2. *Free-text comments* on the 3 and 6-month follow-up questionnaires from women taking part in the *Contraception Choices* RCT (see Box 1).

#### Qualitative interviews

Interviews were conducted by telephone or face-to-face by KB or AG, either at University College London or in clinic settings. We used a semi-structured topic guide to explore participants' views on the online trial design, their views of the *Contraception Choices* website, and digital interventions for contraception. The topic guide covered reasons for taking part in the study, understanding of the purpose of the research, online registration and consent process, acceptability of contact/follow-up via email, views about incentives and the *Contraception Choices* website (e.g. preference for an access point, relevance and usefulness of the website). We tried to mitigate social desirability bias by asking open questions and encouraging participants to express their views, whether positive or negative. Interviews were audio-recorded and transcribed verbatim.^14^ KB was an independent researcher, whilst AG was involved in the *Contraception Choices* website development. Participants were offered £5 online shopping vouchers for completing the 1-week follow-up questionnaire and £15 for taking part in a post-study interview.

#### Free-text comments on RCT follow-up surveys

Participants in the main *Contraception Choices* RCT were followed up by email 3 and 6-months post-randomisation and invited to complete an online outcome questionnaire. For the present study, we analysed participants' answers to two free-text questions: ‘What did you like or dislike about the website’ (asked to intervention group participants only) and ‘Has being in the study had any good or bad effects on your life?’ (asked to all trial participants).

### Data analyses

#### Qualitative interview data

Interviews were audio-recorded, transcribed verbatim and checked for accuracy. The quality of interview data was reviewed by the research group as the study progressed by listening to audio-recordings and discussion of two early transcripts. Following this, the topic guide was revised in minor ways. Data were analysed using thematic analysis, using both deductive and inductive approaches.^15^ KB and AG independently coded interview data, using Atlas.ti™ software to facilitate data retrieval, coding and linkage, and to record analytic notes.

#### Free-text comments

Free-text comments on RCT follow-up surveys were collated and coded in an Excel file, and analytic notes were added. AG and JSh coded the free-text data.

### Data synthesis

A coding frame was derived from the analysis of the interview data, and was refined in light of the analysis of free-text comments. Coding decisions were reviewed by JB, and data analysis meetings were held with the wider research team to discuss the coding frame, emergent themes and interpretations.^15^ Findings from the interviews were concordant with the themes derived from the free-text comments.

## Results

### Participant characteristics

#### Pre-trial feasibility study participants

Twenty-eight women were recruited to the online pre-trial feasibility study. Of these, 18 agreed to be interviewed, seven declined and three were uncontactable. We do not know the number of potentially eligible participants in the six research settings or the number who declined to participate. Participants were interviewed from all six settings (community pharmacy, *n* = 4; antenatal clinic, *n* = 2; abortion clinic, *n* = 3; young person's sexual health clinic, *n* = 4; sexual health clinic, *n* = 3; general practice, *n* = 2).

Participants ranged in age from 17 to 34 years. One participant over the age of 30 years had joined the study despite the age of 15–30 years eligibility requirement. Two women were currently pregnant, six were not using contraception and were not wanting to conceive, and 10 were using at least one method of contraception. Most women were of White ethnicity and were educated to at least A/AS level. Characteristics of participants recruited to the pre-trial feasibility study are shown in Table 1.

**Table 1.** Demographic characteristics of interview participants.

	*n*
Recruitment setting	
General practice	2
Sexual health clinic	3
Antental clinic	2
Abortion service	3
Community pharmacy	4
Young person's sexual health service	4
Age (years)	
17–24	12
25–34	6
Ethnicity	
White	14
Mixed	1
Asian, Asian British	1
Black, Black British	2
Highest level of education	
Degree level	7
Diploma in higher education	1
A/AS levels	9
General Certificate of Secondary Education (GCSE)	0
Other qualifications	1
Contraception method used	
None (currently pregnant)	2
None (wanting to conceive)	0
None (not wanting to conceive)	6
Withdrawal or fertility awareness	1
Condoms or diaphragm	1
Pill, patch or ring	8
Long-acting methods	0
Sterilisation	0

#### Contraception choices main RCT participants

The median age of the RCT participants (*n* = 927) was 24 years, with 50% aged 21–27 years.^5^ Most women were of White ethnicity (68%). The remaining participants were of mixed heritage (11%), Asian (9%) and Black (9%). Around half were educated to degree level (51%).

Of the 464 intervention group participants, 387 (83%) provided free-text comments at the 3-month follow-up, and 414 (89%) provided free-text comments at the 6-month follow-up.

### Women's views of participating in the *Contraception Choices* trial

There were five main themes from the qualitative interviews and free-text comments: (1) being approached about the study in a clinic waiting room, (2) attitudes towards participating in contraception research, (3) views of online trial procedures, (4) views of electronic follow-up procedures (email, text messages) and (5) the place of digital interventions for contraception choice.

#### Being approached about the study in a clinic waiting room

The majority of participants felt it was appropriate and acceptable to have been approached about the study in the waiting area of a clinic or pharmacy:

I thought that was good, because it gives you something to do when you’re just sitting there waiting, rather than just worrying about what's going on or anything. It comes as a nice distraction to be honest. (20 years, young person's sexual health clinic, interviewed)

A couple of participants thought that clinic-based recruitment could be sensitive for some:

I think some people would’ve found that uncomfortable to talk about, yes. Some people, when they go to these clinics, just want complete, like want to be really anonymous. (20 years, young person's sexual health clinic, interviewed)

Being invited to participate in contraception research prior to an abortion was problematic because of the stigma connected with attending an abortion clinic, and feeling distracted and concerned by the procedure itself. One participant said:

It was quite embarrassing being there and then I think being approached by two people you don't know is – I don't know how to say it I guess it's like, because I appreciate what you’re doing […..] But I think for some people that would just be quite maybe like I use the word offensive – I don't mean that it's offensive because I don't find it offensive, but I think maybe some people would, yes.

If you try to get information out of people on the day your mind is just not on that subject like you’re not thinking about somebody else's research study. You’re thinking about oh my God am I going to die? (26 years, abortion clinic, interviewed)

#### Attitudes towards participating in contraception research

The perceived credentials of the research team were important in establishing trust (i.e. NHS or a university):

It was important that it was someone that I sort of knew. Wasn't just a random person, but someone I could trust with information … if it's the hospital and the university then they’re going to be the best people that know most about it, probably. (20 years, young person's sexual health clinic, interviewed)

Most participants found it unproblematic and appropriate being asked about contraception and pregnancy in the online survey*:*

I don't feel uncomfortable talking about contraceptives … Whereas people might feel more uncomfortable talking about their sexual life in itself, where contraceptive is kind of, yes, nothing embarrassing. (23 years, community pharmacy, interviewed)

In contrast, one participant recruited from the abortion service felt ambivalent about being asked about contraception and pregnancy at that time:

I don't really know I think I was quite mixed there because I was definitely like not, well I guess I was just like cursing myself at the time I was like this is something I should have thought about before. (26 years, abortion clinic, interviewed)

Several women said that the research sounded interesting and that they wanted information about contraception and felt they may learn more by seeing the *Contraception Choices* website. A number of participants wanted to help others by contributing to research:

I feel really proud of myself for contributing to improving health information in a small way. Family planning/contraception are health issues I really care about – it's nice to ‘give back’. It's great that I’ve been able to contribute. It has made me feel like I’m doing something worthy. (30 years, sexual health clinic, free-text comment)

Some women felt that the voucher was an incentive to take part in the study and a nice reward for their time, but for others, the voucher was not important. All but one participant said that they would have taken part in the research even if a voucher was not offered:

The questionnaire isn't invasive or lengthy and the voucher is nice little incentive to take part. (27 years, general practice, free-text comment)

#### Views of online trial procedures

Online trial registration was self-directed using a tablet computer in clinic waiting rooms. Women found it quick and easy to register for the study and found the information clear, such that they largely understood what the study was about and what they were agreeing to. Women did not have any concerns about giving their contact details (telephone number and email address) and understood these were so the research team could send them the follow-up questionnaire and to contact them to arrange an interview:

… the information I gave I felt was standard for what I was about to participate in. It was what you would expect when signing up to anything online … (21 years, community pharmacy, interviewed)

… it's a kind of tablet [computer] I haven't used before. But despite that, it, because tablets are made quite user friendly and the questionnaire was very user-friendly, so even for me, never seeing that kind of tablet before, that was very easy [……] you can sit with a tablet in a way that actually achieves more privacy than if you had a computer you had to go over to. (23 years, community pharmacy, interviewed)

#### Views of electronic follow-up procedures

Participants were followed up by email with a weblink to the online follow-up questionnaire. Non-responders were also contacted by text message and by phone, if needed. Participants were positive about being contacted by email for the follow-up survey because of the convenience:

… it's less personal … if people feel a certain type of way about it … it's easy. It comes in. Boom. It's on my phone. It's on my laptop. So it's quick. It's accessible. So it's probably the best way. (27 years, community pharmacy, interviewed)

… I like being contacted by email because it gives me a lot of … I can kind of control when I have the time for it … So I think it's a good way at least for surveys, etc., where it doesn't, you don't have to respond now. You can respond tomorrow or the day after, if that's when you got time, so, yes, emails are good. (23 years, community pharmacy, interviewed)

One participant said that she liked being contacted by email since she could immediately see who the email was from. While none of the participants minded receiving the follow-up survey by email, some felt that text messages might be more likely to be read:

With emails, I tend to just skim through them very quickly. So I might not pay attention to the content in the email … And with texts … People use texts … People text often, so you, kind of … You’re most likely to view the message than just leave it there. (19 years, young person's sexual health clinic, interviewed)

#### Email reminders to engage with the *Contraception Choices website*

The main trial intervention group were sent emails every two weeks to encourage them to revisit the *Contraception Choices* website, and these prompts were mostly positively received:

If anything the emails have been a helpful reminder to sort a more permanent solution for my contraception needs. (22 years, sexual health clinic, free-text comment)

A minority of participants felt that there were too many prompts to visit the website (although every reminder email offered a link to ‘unsubscribe’ if unwanted).

#### Adverse impacts of the research or the *Contraception Choices website*

One person recruited through the abortion clinic described an adverse impact of being in the study:

I was asked to do the survey on the day of my abortion so the timing was not ideal and emails about this survey remind me of that day. (23 years, abortion clinic, free-text comment)

There were no other reported adverse impacts concerning either the research or the *Contraception Choices* website.

#### The place of digital interventions for *Contraception Choice*

Participants were positive about the *Contraception Choices* website and suggested a number of places where they felt the website could be offered, including healthcare settings such as general practice, hospitals, antenatal or post-natal clinics, pharmacies, sexual health clinics and walk-in centres. Participants also felt the website would be useful in schools, colleges and universities, on social media (e.g. Facebook and Twitter), and also advertised on public transport and in women's magazines.

I think it's very useful, because it gives you so much information than you’re [not] going to get from anywhere else, and especially is all in one place that it's really accessible and easy to understand. (20 years, young person's sexual health clinic, interviewed)

Some said that a website would be more attractive than leaflets:

I think it would be very useful. Last year I had to go several times to a walk-in clinic … in the office I was in there was, maybe, like, pamphlets, but, like, who actually goes through, like brochures nowadays? (21 years, community pharmacy, interviewed)

One participant felt that a website was useful because it meant people could access information without having to speak to someone:

… it's a way that you can access information without asking or speaking to people, I think that people so often just don't want to do that. (26 years, abortion clinic, interviewed)

Participants had differing opinions on when the website would be most useful: before, during or after a clinic appointment. Some suggested that it would be useful to receive a link to the website when making an appointment or along with text message appointment reminders:

… when you were making an appointment and they asked you what it was about maybe being redirected to the website then would have been really good. (19 years, abortion clinic, interviewed)

… I know that we get, from my sex health clinic, text messages about our appointments. If that could include, if I’m female, a link to that, that would be, I’m thinking excellent. (23 years, community pharmacy, interviewed)

Many participants said that the waiting room of a sexual health clinic or antenatal clinic was a good time to offer web-based information on contraception:

I think [it was] a really good idea […] in the waiting room, especially in the antenatal part because that is the time when women would be thinking about, maybe, when they give birth, what to go on and all their options. (22 years, antenatal clinic, interviewed).

Participants also suggested that the *Contraception Choices* website could be a useful tool for clinicians to share in a consultation:

I think it would be a good thing for the nurse to show, like, you know, well, yes, look here, so here we have all, instead of just talking, it's good to have it written down and then I think the website's great. (22 years, young person's sexual health clinic, interviewed).

Some women thought the website would be useful after a contraception appointment, to look-up information another time and to review options following emergency contraception:

… sometimes, when you leave the clinic, you’ve, kind of, forgotten, or you don't … You’re not really clear on everything that you’ve been told by the doctor. So it just … it allows you to, kind of, brush up on any, like knowledge. (19 years, young person's sexual health clinic, interviewed)

… if you’re going to a sexual health clinic for a morning after pill or anywhere for a morning after pill if they like talked to you about that, about this website then that would be really useful. (19 years, abortion clinic, interviewed)

## Discussion

Our findings show that young women who participated in online contraception research appreciated the convenience of the online research design and expressed no major concerns about self-registration on a tablet computer, online informed consent, online data collection, privacy and data security or being followed up by email or text message.

Young women valued the research topic (contraception), and placed trust in research which was run by a university in collaboration with the NHS. Women generally did not mind being approached about the study in clinic waiting rooms, although the topic and timing were more problematic in the abortion clinic setting.

Women found the *Contraception Choices* website engaging and helpful and were keen to see it offered in sexual health and community clinic waiting rooms and elsewhere, as a supplement to clinical services.^4^ This qualitative research confirms the feasibility and acceptability of online trial procedures for contraception research, and affirms the need and appetite for accurate information and tailored help with contraception decisions.^16^

### Strengths and limitations

There is a potential selection bias since the study did not capture the views of those who declined to participate in the feasibility study or the main trial, and we interviewed only 18 out of 28 feasibility study participants, so those holding negative views of the research topic and/or trial design may well be under-represented. We interviewed small numbers from each clinical setting, which allowed us to assess user views of the trial design but limits comparisons between settings.

The study was a pragmatic process evaluation that addressed questions about the proposed RCT study design and the feasibility of a digital intervention for contraception in different settings, so a thematic content analysis was appropriate.^17^ Some interviews were very short because of time pressure in the clinic settings and free-text comments on the RCT follow-up questionnaires were optional and mostly very brief, so we could not explore participant views in depth. However, themes within the brief free-text comments were consistent with themes derived from interviews.

### Implications of findings for trial design

#### Recruitment

Online recruitment to online trials can be quick and easy, for example, via social media^18^ but face-to-face recruitment in clinic settings can enhance participation in digital health research.^11,12^ We recruited participants face-to-face for the main *Contraception Choices* RCT, and also via an online booking system (sending an invitation within a text message confirming a contraception appointment).^5^ Automated online recruitment was much quicker and less resource-intensive than face-to-face recruitment and is highly appropriate in the context of the coronavirus pandemic.^19^ The credentials of a research team are important to participants,^20^ and should feature on online recruitment adverts.

Recruitment in the abortion clinic setting was more difficult for participants who may experience negative feelings such as guilt, shame or self-blame,^21^ and who may feel distracted and concerned about the procedure itself.

#### Online trial procedures

Online questionnaires and online trials are now very well-established methods for sexual health research, including national surveys of sexual behaviour, interventions for sexually transmitted infection (STI) and human immunodeficiency virus (HIV) prevention and for sexual difficulties, and sex and relationship education.^7,9,12,18,22–24^ Our research confirms that participants are not concerned about an online environment for sexual health research as long as they are given information about what is involved and they trust the research team.^20,25^ Participants can be offered a choice of mode of contact (e.g. by email, phone, text message and/or post).^12^

Online sexual health research could incur unanticipated harms, for example, if others glean private information from study emails or text messages.^26^ Mobile phone use is not private in many situations, for example, phones may be shared within families in low-income countries^26,27^ and friends, parents or partners may see young people's phone content.^28,29^ Abusive partners may exert control through violation of digital privacy (e.g. monitoring emails, social media accounts and text messages).^26,28^ It is reassuring that none of the RCT participants or pre-trial feasibility study interviewees reported adverse events of this nature. It is important to strike a balance between describing research procedures in enough detail so that participants can evaluate potential risks and ensuring that information is clear, concise and easy to understand.^13,30^

### Interactive digital interventions for contraception choice

Interactive digital interventions (IDIs) are effective for sexual health and HIV knowledge and behaviour change.^31,32^ IDI can increase contraception knowledge,^33^ uptake of more effective contraceptive methods and contraception adherence,^34–36^ and decrease unplanned pregnancy.^37^ IDIs can be expensive to develop, but offer the advantages of content accuracy and fidelity, and the potential to reach large audiences with relatively low dissemination costs.^38^ However, despite patient enthusiasm for online sexual health resources,^30^ healthcare professionals' reservations can be a barrier to implementation.^39^

Young people are more likely than older age groups to seek sexual health information online^40^ and digital interventions such as the *Contraception Choices* website can meet a need for trustworthy information and support for decision making.^16,30^ Digital interventions can complement face-to-face health services in a variety of ways, for example before consultations^36^, in consultation (to facilitate informed choice discussions^41^) and after consultations (e.g. via mobile phone^42^). Provider-initiated conversations regarding abortion and contraception can be experienced as judgemental and/or coercive^43,44^ and in this context, digital decision aids which offer a full range of contraceptive methods and adequate time for deliberation may offer a greater sense of autonomy.^43^

## Conclusions

Women appreciated the advantages of the online research design and did not express concerns about online consent, privacy, follow-up by email or text or contraception and fertility as topics, although the waiting room of an abortion clinic was a less acceptable setting than other health services (sexual health clinics, antenatal clinics, community pharmacy and general practice). Participants felt that digital interventions for contraception would be useful in a variety of settings. Sexual health services are increasingly offered online^1,45^ and digital resources for health promotion and contraceptive decision making should be a natural complement to online services such as STI testing and online contraception provision.
